# Physicians’ Awareness of Depression Among Their Patients in Saudi Arabia

**DOI:** 10.7759/cureus.28742

**Published:** 2022-09-03

**Authors:** Anwar A Sayed, Jennifer NW Lim, Kelly McFarlane

**Affiliations:** 1 Department of Surgery and Cancer, Imperial College London, London, GBR; 2 Department of Medical Microbiology and Immunology, Taibah University, Madinah, SAU; 3 Institute of Health, University of Wolverhampton, Wolverhampton, GBR; 4 Department of Clinical Medical Sciences, University of the West Indies, Champs Fleurs, TTO

**Keywords:** saudi arabia, physicians’ knowledge, physicians’ attitude, mental health, depression

## Abstract

Background

Depression is a global public health burden, and although it is multifactorial, its development is highly associated with chronic diseases. Thus, physicians’ knowledge and attitude toward depression are vital for the recognition and treatment of depression among patients with chronic illnesses. This study aims to examine physicians’ knowledge and attitude toward depression in Saudi Arabia and to determine factors that significantly influence them.

Methodology

An online survey using a 12-item questionnaire was completed by 50 physicians between January and June 2020. Knowledge (K) and attitude (A) scores were calculated and compared nonparametrically based on scores by gender and years of experience.

Results

Participants’ years of clinical experience was found to significantly influence the participants’ K scores, with those with fewer than one year of experience having the highest K scores of all participants (p < 0.05). Years of experience had a similar influence on the A scores. Furthermore, gender was an influencing factor as male participants had higher A scores than female participants. Years of experience and gender influenced the A scores independently. Male physicians more commonly referred patients to a mental health specialist than female physicians, and had significantly higher A scores and more years of experience. There was a direct correlation between the K and A score, indicating that participants’ knowledge of depression positively influenced their attitude toward depression.

Conclusions

In this study, participants’ gender and years of experience have been identified to have a significant impact on their knowledge and attitude toward comorbid depression when treating patients with chronic illnesses. This identifies an important gap and offers preliminary insight into the readiness and practice of holistic care for patients, particularly for those treated by physicians of differing gender and experience levels. Findings further demonstrate that it is most beneficial to patients with chronic illnesses that physicians utilize a holistic approach and consider depression when developing their treatment plans. This would involve being able to detect and manage depression among their patients properly, as well as referring patients to mental health specialists when needed. Clinical guidelines should be updated to emphasize the use of depression screening tools for patients with chronic diseases.

## Introduction

Depression is a global problem that knows no border. Globally, the prevalence of depression has been estimated to exceed 260 million cases [1]. In Saudi Arabia (SA), the reported prevalence of depression ranged between 23% and 49% [2-5]. Depression is reported to be consistently linked to the presence of comorbidities; this condition is highly prevalent among patients with type 2 diabetes mellitus, sickle cell disease, and chronic renal failure requiring hemodialysis [2-5]. Elderly people in SA were 3.15 times more likely to suffer from depression than younger people mainly due to the presence of comorbidities [6]. Other factors that could have contributed to these results include certain social determinants of health such as poverty, living in isolated and rural areas, and lack of access to medical services [6-8]. Being female was also found to be positively correlated with the experience of depression in SA [6,9].

Dealing with depression is neither a straightforward nor an easy task and has always been tangled with challenges. Many social challenges have been identified and addressed extensively in the literature across different populations. One of these challenges is the stigma associated with depression, which prevents patients from seeking and continuing treatment [10,11].

Medical challenges also constitute a major obstacle in various stages of managing depression, namely, access to services, detection, and effective treatment. Two such challenges include the attitudes and perceptions of physicians toward mental health and depression. These physician-related issues represent a potentially major yet frequently overlooked obstacle that further “adds salt to the wound” when dealing with depression.

International and local guidelines in SA recommend regular screening for depression for all adults, especially those with comorbidities, a group considered to be at high risk [12-14]. Despite the guidelines, depression is significantly underdiagnosed in SA. Physicians in SA do not seem to follow the current guidelines for using depression screening tools. This has been noted, for example, in the use of the Patient Health Questionnaire (PHQ) and the use of the recommended multidisciplinary approach, which involves referring patients to mental health specialists when needed [15]. The current practice of managing depression, either in primary, secondary, or tertiary healthcare settings, is also ineffective and does not meet the evidence-based criteria, regardless of its medical or psychological nature [7,16].

In SA, to date, only two studies have attempted to explore depression from a physician’s perspective. Al-Atram examined the knowledge of over 140 physicians (general practitioners, specialists, and family physicians) about depression and found that over half of them were capable of identifying symptoms of depression. Furthermore, most of them reported that detecting depression among elderly patients as well as assessing its severity are common issues [17]. Aldahmashi and colleagues explored physicians’ perceptions of depression in a qualitative study and found that the majority of physicians preferred dealing with physical illnesses rather than mental ones [18]. These two studies focused primarily on physicians, regardless of their specialties, who were working at secondary and tertiary healthcare facilities. Although they addressed the physicians’ knowledge and attitude separately, the authors did not examine the relationships between physicians’ knowledge and attitude toward depression or social determinants, such as gender, clinical experience, and physicians’ specialties, which may influence knowledge and attitude.

This paper attempts to bridge the research gap between the findings of physicians’ knowledge and attitude toward depression and the factors influencing their knowledge and attitude towards depression.

## Materials and methods

Study design and data collection plan

This is a cross-sectional study in which a self-report voluntary online survey was conducted to assess physicians’ knowledge and attitudes toward depression among their patients. The questionnaire has been devised and adopted from a previously validated questionnaire [19]. It was distributed via the Saudi Ministry of Health online portal, professional emails, and social media platforms (Facebook and WhatsApp). The questionnaire comprises four main sections, namely, participant characteristics, medical education and experience of current practice, knowledge assessment of depression, and attitude (Appendices). Knowledge (K) and attitude (A) scores were calculated based on the responses provided. Each response was given a score of either 2, 1, or 0 based on its positivity (not likely = 0, likely = 1, very likely = 2). The survey was conducted following the guidance on the medical use of social media [20].

The sampling strategy

The study included only physicians, on a random sampling, who are actively treating patients and excluded physicians who have retired or work non-clinically. Allied health workers such as nurses and technicians were also excluded from the study. As English is the official language of the medical practice in SA, those who could not read English were excluded from this study.

It was estimated in 2019 that the number of physicians in SA who would be eligible for participation in this study would reach almost 2,500 doctors [21]. The sample size, based on a 95% confidence level and a 10% margin of error, was estimated to be 93 participants [22]. The questionnaire was disseminated to over 250 physicians between January 2020 and June 2020; a period coinciding with the coronavirus disease 2019 (COVID-19) pandemic. Despite the numerous attempts made to encourage participation, only 50 physicians completed the survey resulting in a response rate of 20%.

Statistical analysis

The chi-square test was used to compare between collected categorical data regarding several aspects of physicians’ awareness of depression across certain variabilities such as gender, job title, and type of workplace [23].

The distribution of the numerical datasets was determined using the Shapiro-Wilk test. Parametric and nonparametric methods were used on datasets with normal (Gaussian) and non-normal distribution, respectively. Unpaired Student’s t-test and Mann-Whitney U tests were used to compare two parametric and nonparametric datasets, respectively. To compare more than two groups of data, one-way analysis of variance (ANOVA) with Bonferroni correction for multiple comparisons was used for parametric datasets. For nonparametric datasets, the Kruskal-Wallis test with Dunn’s correction for multiple comparisons was used.

Correlations between the calculated scores (knowledge and attitude) and physicians’ age and years of experience were determined using a two-tailed Pearson’s correlation coefficient. Statistical significance was denoted at a p-value less than 0.05. Data analysis was carried out using GraphPad Prism version 8.4 (GraphPad Software, California, United States).

Ethical considerations

Ethical approval was obtained from the University of Wolverhampton, Faculty of Education, Health and Wellbeing Research Ethics Committee, and Taibah University College of Medicine Research Ethics Committee (approval number: 010-1441). Participants were required to consent to the participation at the start of the online survey.

## Results

Participants’ characteristics

The majority of the 50 participants were males (78%). This disproportion in the male-to-female ratio was because the number of male physicians is more than twice the number of female physicians in SA [24]. The median age of the participants was 28 years (interquartile range = 26-33.50). The characteristics of the participants and their clinical experience are summarized in Table 1.

**Table 1:** Characteristics of the study’s participants. *Years are expressed as a median. ^Healthcare establishments in Saudi Arabia. IQR: interquartile range

Characteristics	Answers, n (%)
Gender	Male: 39 (78%)
	Female: 11 (22%)
Age	28 years old* (IQR = 24–63)
Specialty	Family medicine: 10 (20%)
	Internal medicine: 10 (20%)
	Miscellaneous: 7 (14%)
	Obstetrics and gynecology: 6 (12%)
	General surgery: 5 (10%)
	Orthopedics: 5 (10%)
	Radiology: 3 (6%)
	Emergency medicine: 2 (4%)
	Pediatrics: 2 (4%)
Job title	Intern: 1 (2%)
	General practitioner: 12 (24%)
	Resident: 17 (34%)
	Registrar/Specialist: 10 (20%)
	Consultant: 10 (29%)
Place of work^	Primary healthcare center: 5 (10%)
	Secondary hospital: 31 (62%)
	Tertiary hospital: 14 (28%)
Years of experience	1.5 years* (range = 0.1–40 years)
The country where medicine was studied	Saudi Arabia: 43 (86%)
	Middle East: 7 (14%)
The country where medical internship/speciality training was completed	Saudi Arabia: 41 (82%)
	Middle East: 5 (10%)
	Europe: 2 (4%)
	Other: 2 (4%)
Average number of patients seen daily	<10 patients: 9 (18%)
	10–30 patients: 35 (70%)
	31–50 patients: 6 (12%)
Number of patients referred to psychiatrist in the last 3 years	None: 23 (46%)
	1–3 patients: 14 (28%)
	4–10 patients: 8 (16%)
	>10 patients: 5 (10%)
Attendance of mental health training/workshop/seminar in the last 3 years	Yes: 16 (32%)
	No: 34 (68%)

The participants of this study were equally distributed between medical and surgical specialties, and the majority of them studied medicine and completed their medical training in SA. Almost half of the participants did not refer a patient to a psychiatrist, and the majority did not attend any mental health training in the last three years.

Influence of years of experience on knowledge and attitudes toward depression

The knowledge (K) scores achieved by participating physicians, ranged between 0 and 14, with a median score of 8. No other participants’ characteristics were found to significantly influence their K scores.

**Figure 1:**
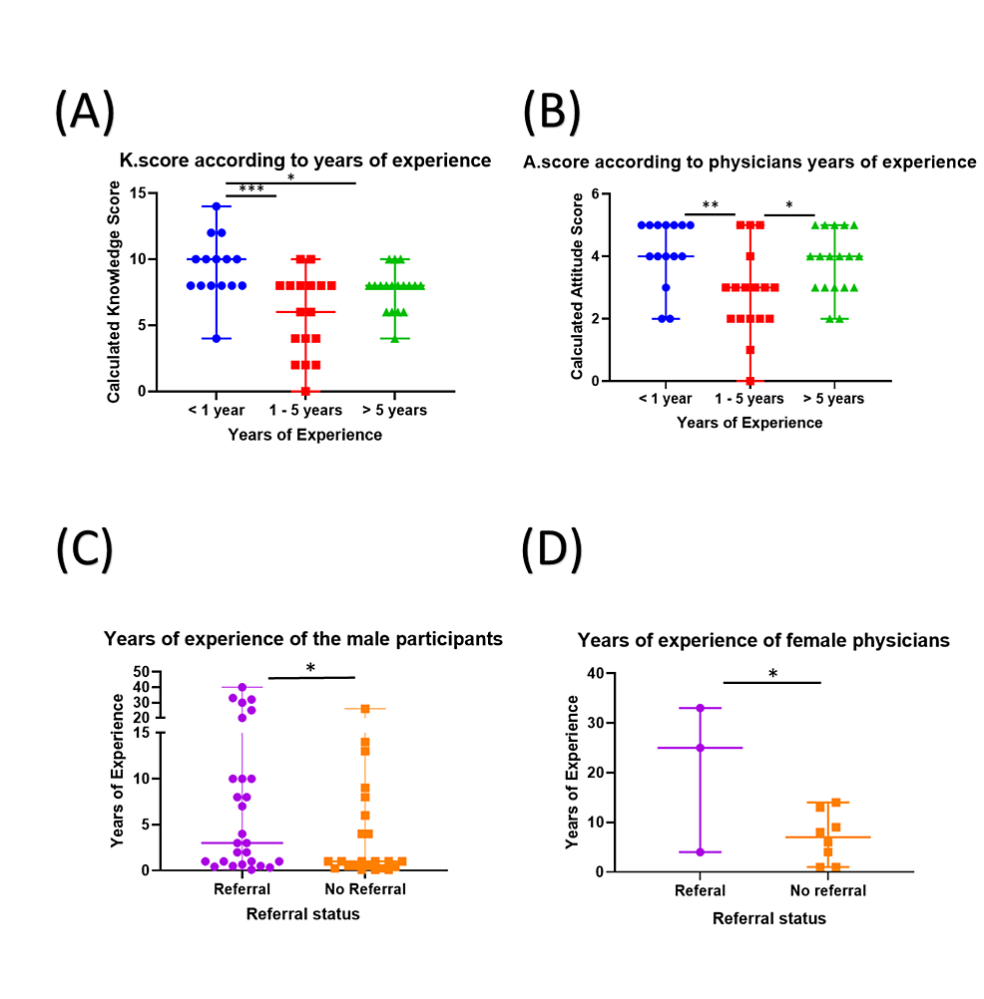
Comparisons of K and A scores, as well as the participating physicians’ referrals according to their years of experience. (A) Comparison of K scores of the participants based on their duration of clinical experience. Physicians with less than one year of experience (blue circles) had the highest K scores compared to those with one to five years of experience (red squares) and those with over five years of experience (green triangles). (B) A comparison of A scores of the participants based on their years of experience. Both participants with less than one year of experience and those with over five years of experience had significantly higher A scores compared to those with one to five years of experience. (C) Comparison of clinical experience between male participants based on their referral status. The figure shows that participants who previously referred patients for mental healthcare (purple; n = 24) have significantly more experience compared to those who did not (orange; n = 15). (D) Comparison of the clinical experience of female participants based on their referral status. Female participants who previously referred patients had significantly longer clinical experience compared to those who did not. K score: knowledge score; A score: attitude score; *: p-value < 0.05; **: p-value < 0.01; ***: p-value < 0.001

Physicians were categorized into the following three groups based on their years of experience: those with less than one year of experience, those with one to five years of experience, and those with more than five years of experience. The rationale for this grouping was based on the clinical training experience in SA. Interestingly, those with less than one year of experience had the highest K scores compared to those who had between one and five years (10 vs. 6; p = 0.001) and more than five years of experience (10 vs. 8; p < 0.05). Those who had more than five years of experience had higher K scores compared to those with one to five years of experience; however, this difference was not statistically significant (8 vs. 6; p = 0.35) (Figure 1, Panel A).

The participants’ A scores differed significantly based on their years of experience. Those with one to five years of experience had the lowest A scores compared to the other two groups. Those with less than one year of experience had significantly higher A scores compared to those who had between one and five years of experience (4 vs. 3; p < 0.01). Participants with more than five years of experience also had significantly higher A scores compared to those with one to five years of experience (4 vs 3; p < 0.05) (Figure 1, Panel B). A two-way ANOVA test demonstrated that the A scores were significantly affected by both years of experience and gender, with no significant interaction between these two independent factors. No other characteristics were found to significantly influence the A scores.

An important factor relative to participants’ attitudes about depression was whether they had previously referred a patient to a mental health specialist. About 27% of the female participants (n = 3) previously referred patients in the past three years whereas 61.5% of the male participants did. The participants who previously referred patients had significantly longer experience compared to those who did not (p < 0.05) (Figure 1, Panel C-D).

Influence of gender on knowledge and attitude toward depression

**Figure 2:**
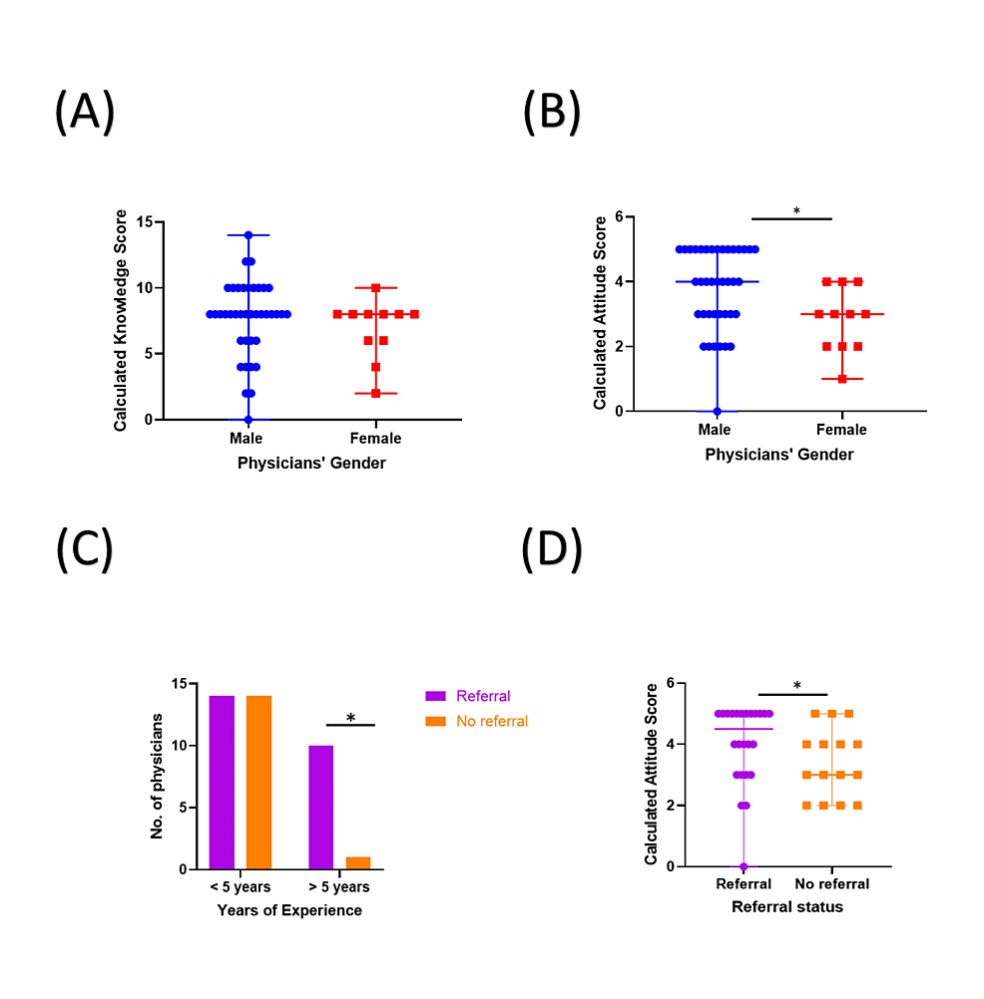
Influence of gender on participants’ knowledge and attitudes. (A) The K scores of the participants were comparable between male (blue circles) and female (red squares) physicians. (B) Comparison of the A scores of male participants was significantly higher compared to female participants. (C) The bar chart demonstrates the distribution of male participants based on their referral status as well as the duration of their experience. The chart demonstrates that the proportion of male physicians who refer patients (purple) is significantly higher among those with more than five years of experience compared to those with less than five years of experience. (D) A comparison of the A scores of male participants according to their referral status. The figure demonstrates significantly higher A scores for participants who referred (purple) compared to those who did not (orange). A score: attitude score; *: p-value < 0.05.

Next, we assessed whether the gender of participants affected their knowledge (K) score. Male and female participants had comparable K scores with no significant difference between them (p > 0.05; Figure 2, Panel A). The attitudes (A) score of the participants ranged between 0 and 5 with a median score of 4 (out of 5). Gender seemed to influence the A scores as male participants had significantly higher A scores compared to female participants (4 vs. 3; p < 0.05) (Figure 2, Panel B).

Among male participants, the duration of clinical experience appeared to be an influencing factor in their referring patients. Furthermore, applying Fisher’s exact test demonstrated that the proportion of male physicians who referred patients was significantly higher among those with more than five years of experience compared to those with less than five years of experience (90.9% vs. 50%; p < 0.05) (Figure 2, Panel C).

Male participants who previously referred a patient had significantly higher A scores compared to those who did not (5 vs. 3; p < 0.05) (Figure 2, Panel D). Interestingly, the A scores were comparable between female participants who previously referred patients and those who did not.

Interaction of K and A scores

**Figure 3:**
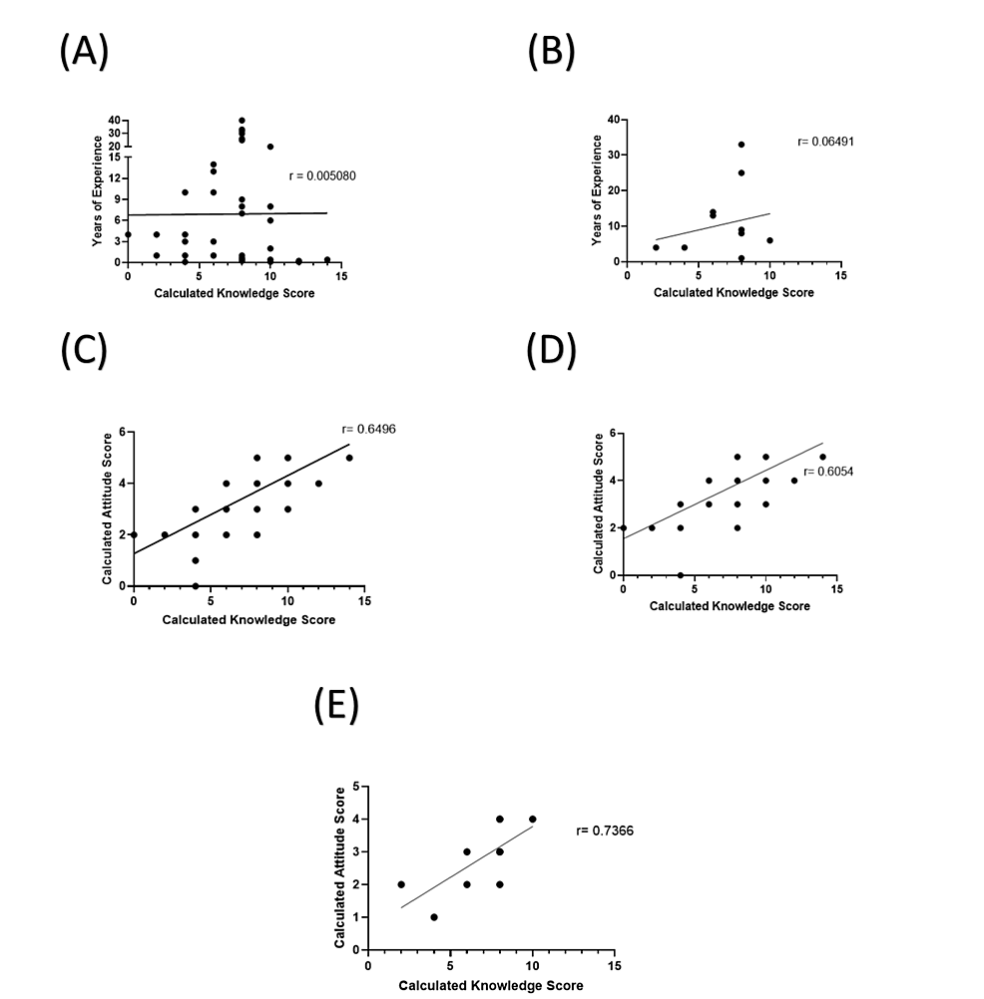
Correlations between the K scores, A scores, and years of experience of the participants. (A) An indirect correlation between the K scores of the male participants and their years of experience. (B) The direct correlation between the K scores of the female participants and their years of experience. (C) The direct correlation between the K and A scores of the participants. A direct correlation between the K and A scores of the (D) male participants and (E) female participants. K score: knowledge score; A score: attitude score; *: p-value < 0.05; ****: p-value < 0.0001

The lack of direct correlation between the scores and years of experience has highlighted the possibility of an additional factor that may also influence these scores. The comparison of A scores based on gender demonstrated a significant difference. Therefore, the correlation between participants’ scores and their years of experience was divided based on their gender. Among male participants, there was a significant indirect correlation between the K scores and their years of experience (r = -0.34; p < 0.05) (Figure 3, Panel A).

On the other hand, there was a direct correlation, although not statistically significant, between the K scores of the female participants and their years of experience (p > 0.05) (Figure 3, Panel B). There was a positive correlation between the years of experience and A scores of male and female participants; however, neither was statistically significant.

The next task was to determine whether there was an association between participants’ knowledge of depression and their attitudes toward it. The calculated K scores of the participants directly correlated with their calculated A scores, and this correlation was statistically significant (r = 0.64; p < 0.0001) (Figure 3, Panel C). Similar positive correlations were found between the K and A scores of male (r = 0.6; p < 0.0001) and female participants (r = 0.73; p < 0.05) (Figure 3, Panel D-E). These positive correlations indicate a direct relationship between the K and A scores of the participants. In other words, the higher the K score of participants, the more likely they will have a higher A score.

## Discussion

This is the second study to address physicians’ knowledge of depression in SA. The strengths of this study lie in the variable participants’ demographics and the use of numerical values to establish a link between participants’ knowledge and attitude. To further facilitate the assessment and comparison of the participants’ knowledge and attitude according to their characteristics, knowledge score (K score) and attitude score (A score) have been developed. Overall, the variation in the participants’ knowledge of depression, as measured by the K score, showed a normal distribution, with the majority of participants scoring 7-8 (out of 14). K scores did not differ significantly between participants based on their gender, place of work, patient load, or job title. Thus, confirming that these factors do not influence physicians’ knowledge of depression. This finding is in line with the work of Liu et al., demonstrating that the gender and specialty of the participants did not affect their knowledge of depression [25].

Additionally, participants’ K scores did not differ significantly based on their specialty, in line with previous studies. Gallo and colleagues reported the lack of a significant difference in knowledge of depression between family physicians and internists [26]. Similarly, another study including 970 certified family physicians did not observe any significant differences in their knowledge of depression based on gender, age, and clinical experience [27].

The availability of a validated and reliable screening tool should facilitate the detection of depression in any clinical setting. In SA, an Arabic version of the PHQ-9 screening tool for depression has been developed and validated to be used reliably in the primary healthcare setting [28,29]. The Saudi Ministry of Health has issued a national guideline further emphasizing the use of PHQ-9 to detect depression among patients with diabetes [14]. The lack of using depression screening tools by the participants of this study (three out of 50 participants) is in line with previous findings from another study in SA. Albarrak and colleagues found that the ineffective implementation of current guidelines was evident in the lack of official documentation of mental health assessments, that is, whether patients are mentally healthy or in need of a professional mental health intervention [15].

The attitude of physicians toward depression is crucial when dealing with a patient who might suffer from depression. In this study, gender was found to be an independent influencing factor as male physicians had significantly higher A scores compared to female physicians, regardless of their workplace. This finding is in line with a previous cross-sectional study conducted in SA across four tertiary hospitals [18].

Referring patients with suspected depression to a mental health specialist is an important aspect of physicians’ attitudes toward depression. In this study, over 60% of the participants referred patients to a mental health specialist, similar to Al-Atram’s study [17]. Furthermore, patient referral was more frequent among male participants (over 60%) compared to their female counterparts (approximately 28%). Similar results were reported by Heiman and colleagues [30], whereas Becker reported that female physicians were better at dealing with depression [31]. Other studies did not observe differences between male and female physicians’ attitudes toward depression [32,33]. This discrepancy in the findings between studies could be attributed to the job title of the participants.

Physicians who previously referred patients had significantly longer years of experience as well as higher A scores. This finding indicates that the duration of physicians’ clinical practice influences their capacity to refer patients when needed, in line with Spagnolo et al.’s study [34].

The knowledge and attitudes of physicians toward depression have been assessed in several studies. However, these two aspects were not linked. In this study, the development of K and A scores allowed for assessing the link between participants’ knowledge and attitude toward depression. Establishing such a link is necessary to show that the attitude of a person depends on his/her prior knowledge. In this study, a significant positive correlation was observed between K and A scores, indicating a direct relationship between these two elements. In other words, a physician with good knowledge of depression, that is, has a high K score, is expected to have a positive attitude toward depression, that is, a high A score. This important finding is in line with a study by Manzanera et al. which described a significant improvement in the physicians’ attitude toward depression after training in depression [27]. It is important to consider that the differences in the results of this study and others could be attributed to cultural differences, which is beyond the scope of this study.

The strengths of this study lie in the variable participants’ demographics and the use of numerical values to establish a link between participants’ knowledge and attitude. The development of these numerical tools (K and A scores) facilitated the assessment and comparison of participants’ knowledge and attitude according to their characteristics, such as gender and years of experience. Furthermore, it allowed us to evaluate the correlation between them and assess if they influence each other.

The limitations of this study include the low response rate (20%), although the questionnaire was sent directly to 250 practicing physicians. Such low participation is most likely attributed to the timing of the study as it coincided with the ongoing COVID-19 global pandemic. The demographics of the participants are not similar to the physicians’ community in SA [21]. Ideally, the study should have a male-to-female ratio of approximately 1:1, and non-Saudi participants should represent at least 50% of the total participants of this study for the results to be reflective of the current practicing physicians in SA. The physicians’ knowledge of depression and their attitude toward were assessed using a questionnaire developed specifically for this study. This limitation prevented this study to be compared like-for-like to other studies. Alternatively, other validated questionnaires could have been used for this study, such as the revised Depression Attitude Questionnaire (R-DAQ) and the MICA scale [35,36], to enable like-for-like comparisons.

Future work should build upon the findings of this study by addressing the limitations of this study. Public health policies should acknowledge the challenges facing physicians in managing their patients with depression from both physicians’ and patients’ perspectives. Clinical guidelines should be issued or updated in case of their availability to emphasize the use of depression screening tools among patients with chronic illnesses.

## Conclusions

Physicians’ knowledge and attitudes toward depression are vital for patient care in SA. In this study, participants’ gender and years of experience have been identified to have a significant impact on their knowledge and attitude. Physicians who are treating patients with chronic diseases are encouraged to adopt a holistic approach toward their patients. This approach includes being able to detect and manage depression among their patients properly, as well as referring patients to mental health specialists when needed. Therefore, clinical training and treatment guidelines should be updated, and mental health training for physicians should be provided in SA.

## Appendices

**Table 2:** The items included in the study questionnaire. The table demonstrates the four main sections of the questionnaire tool used in this study. Each of these sections contained a number of questions described in the table along with their types of answers. MCQ: multiple choice questions

Section	Questions	Type of answer
Participant characteristics	Gender	Single choice MCQ
	Age	Free text
	Specialty	Free text
	Job title	Single choice MCQ
	Place of practice	Single choice MCQ
Medical education and experience of current practice	Country where medicine was studied	Single choice MCQ
	Country where the medical (specialty) training was completed	Single choice MCQ
	Years of experience (as a treating physician)	Free text
	Number of seen daily patients	Single choice MCQ
	Number of patients referred to a psychiatrist/psychologist in the last 3 years	Single choice MCQ
	Attendance of any training/workshop/seminar on mental health problems	Yes/No question
	Discussion of patients’ mental health issues discussed in current practice	Five-item Likert question [from 1 (rarely) to 5 (very frequent]
Knowledge assessment of depression (symptoms, diagnostic protocols, referral system, and treatments)	The likelihood of depression concurrence with chronic illness	Three-item Likert question
	The likelihood of noticing depressive symptoms in patients being treated for chronic illness	
	The suspicion of depression with significant changes in appetite and sleep	Yes/No question
	The suspicion of depression with significant fatigue/lack of energy	
	The commonality of depression among patients with chronic illnesses compared to the general population	Single answer MCQ
	The use of depression screening instruments in current practice	Yes/No question
	The availability of institutional guidance on dealing with mental health disorders	
Assessment of attitude towards depression	The appropriateness of mental wellbeing assessment in current practice	Yes/No question
	The likelihood of referring a patient with a chronic illness to a psychiatrist once depression is suspected	Three-item Likert question
	The importance of including mental health guidance in current practice	Five-item Likert question
	The importance of attending a training/workshop/seminar on mental health problems	Single choice MCQ
	The likelihood of attending a training/workshop/seminar on mental health problems	

Appendix 2: The study tool

A. Characteristics

Gender:

• Male • Female

Age: … years

Specialty: …

1. What best describes your job title?

• Intern            • Resident        • Specialist/Registrar  • Consultant

2. What best describes your place of practice?

• Primary healthcare center    • Secondary hospital   •Tertiary hospital

B. Medical education and experience of current practice

3. Where did you study Medicine?

• Saudi Arabia             • the Middle East  • Europe          • Other: …

4. Where did you complete your medical (specialty) training?

• Saudi Arabia             • the Middle East  • Europe          • Other: …

Years of experience: … years

5. Have you attended any training/workshop/seminar on mental health problems in the last 3 years?

• Yes    • No

6. On average, how many patients do you see every day in your practice:

• <10   • 10-30            • 31-50            • 50+

7. In the last 5 years, how many times have you referred a patient to a psychiatrist/psychologist?

• 0        • 1-3    • 4-10  • >10

8. How frequently are patients’ mental health issues discussed in your practice, e.g., between colleagues, in morning briefs, and clinical meetings?

• 1 (rarely)       • 2        • 3        • 4        • 5 (very common)

C. Knowledge assessment (knowledge of symptoms, diagnostic protocols, referral system, and treatments)

Symptoms:

9. How likely do you think it is that a patient who has a chronic illness would also experience a depressive disorder?

• Not likely     • Likely           • Very likely

10. How likely are you to notice depressive symptoms in patients who are being treated for chronic illness?

• Not likely     • Likely           • Very likely

11. Would you suspect the presence of depression in a patient describing significant changes in appetite and sleep?

• Yes    • No

12. Would you suspect the presence of depression in a patient describing significant fatigue/lack of energy?

• Yes    • No

13. How common do you think mental health problems are among your patients?

• Rare  • Similar to the general population     • very common

Diagnosis:

14. Are you using either PHQ-9, BDI, BDI-II, HDRS, or CES-D screening instruments in your practice?

• Yes    • No

15. In your practice, is there any guidance regarding dealing with patients with mental health disorders?

• Yes    • No

Referral:

16. How likely are you to refer a patient with a chronic illness to a psychiatrist if you suspect that they are experiencing depression?

• Not likely     • Somewhat likely     • Very likely

D. Suggestions and recommendations

17. Do you believe that some level of assessment for mental well-being is appropriate in your practice?

• Yes    • No

18. How important/necessary is it to include mental health guidance in your practice guidelines?

• 1 (not at all)  • 2        • 3        • 4        • 5 (very important)

19. How important is it for you to attend a training/workshop/seminar on mental health problems?

• Not important           • Somewhat important            • Very important            •Not sure/do not know

20. How likely are you to attend a training/workshop/seminar on mental health problems?

• Not likely         • Likely • Very likely       •Not sure/do not know
